# Mitochondrial type II NADH dehydrogenase of *Plasmodium falciparum* (PfNDH2) is dispensable in the asexual blood stages

**DOI:** 10.1371/journal.pone.0214023

**Published:** 2019-04-09

**Authors:** Hangjun Ke, Suresh M. Ganesan, Swati Dass, Joanne M. Morrisey, Sovitj Pou, Aaron Nilsen, Michael K. Riscoe, Michael W. Mather, Akhil B. Vaidya

**Affiliations:** 1 Center for Molecular Parasitology, Department of Microbiology and Immunology, Drexel University College of Medicine, Philadelphia, Pennsylvania, United States of America; 2 Portland VA Medical Center, Portland, Oregon, United States of America; Johns Hopkins University Bloomberg School of Public Health, UNITED STATES

## Abstract

The battle against malaria has been substantially impeded by the recurrence of drug resistance in *Plasmodium falciparum*, the deadliest human malaria parasite. To counter the problem, novel antimalarial drugs are urgently needed, especially those that target unique pathways of the parasite, since they are less likely to have side effects. The mitochondrial type II NADH dehydrogenase (NDH2) of *P*. *falciparum*, PfNDH2 (PF3D7_0915000), has been considered a good prospective antimalarial drug target for over a decade, since malaria parasites lack the conventional multi-subunit NADH dehydrogenase, or Complex I, present in the mammalian mitochondrial electron transport chain (mtETC). Instead, *Plasmodium* parasites contain a single subunit NDH2, which lacks proton pumping activity and is absent in humans. A significant amount of effort has been expended to develop PfNDH2 specific inhibitors, yet the essentiality of PfNDH2 has not been convincingly verified. Herein, we knocked out PfNDH2 in *P*. *falciparum* via a CRISPR/Cas9 mediated approach. Deletion of PfNDH2 does not alter the parasite’s susceptibility to multiple mtETC inhibitors, including atovaquone and ELQ-300. We also show that the antimalarial activity of the fungal NDH2 inhibitor HDQ and its new derivative CK-2-68 is due to inhibition of the parasite cytochrome *bc*_*1*_ complex rather than PfNDH2. These compounds directly inhibit the ubiquinol-cytochrome *c* reductase activity of the malarial *bc*_*1*_ complex. Our results suggest that PfNDH2 is not likely a good antimalarial drug target.

## Introduction

The mitochondrial electron transport chain (mtETC) is an important, validated drug target in malaria parasites. The mtETC is the primary generator of the electrochemical gradient across the mitochondrial inner membrane. In the asexual blood stages of malaria parasites, however, the only critical function of the mtETC is the continuous reoxidation of ubiquinol to sustain activity of DHOD (dihydroorotate dehydrogenase), which is required for *de novo* pyrimidine biosynthesis [1]. In contrast, in insect stages, mitochondrial oxidative phosphorylation appears to have increased importance [2], likely requiring an intact central carbon metabolism [3] and increased mtETC activity to maintain the electrochemical gradient that drives ATP synthesis. For decades, the mtETC of malaria parasites has attracted major drug development efforts [4], ultimately resulting in antimalarials for clinical use and in preclinical/clinical stages of development. Malarone^™^, a combination of atovaquone and proguanil, has been used clinically since 2000. Recent drug development efforts focused on the parasite DHOD led to the clinical candidate DSM265, which is currently undergoing Phase II clinical trials [5, 6]. ELQ-300, an inhibitor of the Qi site of the *bc*_*1*_ complex (Complex III), has also reached preclinical development [7, 8]. This underscores that the essential protein components of the parasite mtETC are attractive antimalarial drug targets.

In the parasite mtETC, there are five dehydrogenases that donate electrons to ubiquinone producing ubiquinol (reduced ubiquinone), including NDH2 (type II NADH dehydrogenase), MQO (malate quinone oxidoreductase), DHOD, G3PDH (glycerol 3-phosphate dehydrogenase), and SDH (succinate dehydrogenase). The reduced ubiquinol is subsequently oxidized back to ubiquinone by the mitochondrial *bc*_*1*_ complex (Complex III). As mentioned above, the parasite DHOD is a validated antimalarial drug target. NDH2 has also been considered a promising antimalarial drug target for over a decade [9–11]. In general, NADH dehydrogenase is a membrane bound flavoenzyme that catalyzes electron transfer from NADH to quinone producing NAD^+^ and quinol. In human mitochondria, a type I NADH dehydrogenase (Complex I) has 45 subunits and pumps protons across the mitochondrial inner membrane concomitant with electron transfer [12]. Mutations of Complex I subunits are responsible for a significant portion of hereditary human respiratory chain disorders [13]. In contrast, malaria parasites lack the conventional multi-subunit Complex I. Instead, they have a type II NADH dehydrogenase (NDH2), which is a single subunit, non-proton pumping protein, likely attaching to the mitochondrial inner membrane and facing the mitochondrial matrix. *Toxoplasma gondii*, another apicomplexan parasite, has two isoforms of NDH2, which both face the mitochondrial matrix, catalyzing oxidation of mitochondrial NADH [14]. NDH2 is also present in bacteria [15], fungi [16] and plants [17], but not in humans or other mammals.

The absence of NDH2 in humans suggests that the parasite enzyme might be a good antimalarial drug target [9–11, 18]. In 1990, Fry and Beesley first measured NADH oxidation activities in isolated mitochondria of malaria parasites (*P*. *yoelii* and *P*. *falciparum*) using two spectrophotometric methods [19]. Briefly, in the first assay, NADH oxidation was coupled to cytochrome *c* reduction and changes of cytochrome *c* absorption spectrum were measured at a wavelength of 550 nm; in the second assay, NADH oxidation produced NAD^+^, directly leading to a reduced absorption at 340 nm. Using these coupled or direct measurements, Fry and Beesley found a robust NADH oxidation activity in mitochondrial samples which was not inhibited by rotenone, a classical Complex I inhibitor. Their data suggested that mitochondria of malaria parasites were able to oxidize NADH and an active NADH dehydrogenase(s) was present. In 2006, Biagini *et al*. also observed significant NADH oxidation activity (direct assay at 340 nm) in total cell extracts of *P*. *falciparum* [9]. Biagini *et al*. used atovaquone and potassium cyanide to block the activities of Complexes III and IV individually, leading to a conclusion that the observed NADH oxidation was due to PfNDH2 [9]. Coincidentally at that time, the ubiquinone analogue HDQ (1-hydroxy-2-dodecyl-4(1H) quinolone) was found to be a potent inhibitor of the fungal NDH2 in *Yarrowia lipolytica* [20]. Later HDQ was shown to be highly effective against *P*. *falciparum* and *T*. *gondii* parasites [10]. Based on these results [9, 10, 18], it became widely accepted that PfNDH2 could be an attractive antimalarial drug target. As a result, a significant drug discovery campaign based on high throughput screening was undertaken to seek HDQ-like inhibitors to specifically inhibit PfNDH2 [21–23], yielding the lead compound, CK-2-68 [22]. Recently, the crystal structure of PfNDH2 was resolved via X-ray crystallization [24], which could further encourage drug development efforts towards PfNDH2 using approaches based on *in silico* docking and structure activity relationships of PfNDH2 and its inhibitors.

The rationale for targeting PfNDH2 or other mtETC dehydrogenases except for DHOD for antimalarial drug development has, however, been controversial [25, 26]. The fact that the entire mtETC in asexual blood stages could be functionally bypassed by expression of the heterologous yeast DHOD from *Saccharomyces cerevisiae* to support pyrimidine biosynthesis in the presence of mtETC inhibition raised the likelihood that PfDHOD is the only essential enzyme among the five mitochondrial dehydrogenases that donate electrons to ubiquinone [1]. The yDHOD transgenic parasites can be grown continuously under a high atovaquone pressure (100 nM) [1, 27]. Under such conditions, the *bc*_*1*_ complex is fully inhibited, which prevents the reoxidation of ubiquinol by the mtETC and, therefore, should block the turnover of all subsequent quinone-dependent dehydrogenases, implying that PfNDH2, as well as PfG3PDH, PfMQO, and PfSDH, are not required for growth. In support of this, the type II NADH dehydrogenase in the rodent malaria parasite *P*. *berghei*, PbNDH2, was genetically ablated in 2011 [28]. Genetic ablation of PbNDH2 halted parasite development only in the mosquito stages, not in the blood stages [28]. Very recently, selection of resistant *P*. *falciparum* parasites by treatment with CK-2-68 and RYL-552, reported “PfNDH2 specific” inhibitors, generated mutations in the mtDNA encoded *cyt b* locus, while no mutations were found in PfNDH2 [29]; these data strongly suggests that CK-2-68 and RYL-552 exert their antimalarial activity by inhibiting the parasite *bc*_*1*_ complex, not PfNDH2, in contrast to previous suggestions [21–23]. Clearly, the idea that PfNDH2 is a promising antimalarial drug target has been challenged with these studies [28, 29]. However, in the absence of specific genetic data on the essentiality of PfNDH2, the importance of PfNDH2 in *P*. *falciparum* has not been settled definitively.

Here, we successfully knocked out PfNDH2 in *P*. *falciparum* using a CRISPR/Cas9 based approach, demonstrating that PfNDH2 is not a good antimalarial drug target. We found that deletion of PfNDH2 did not alter the parasite’s susceptibility to major mtETC inhibitors and, further, that HDQ and CK-2-68 exert their antimalarial activities by directly inhibiting the parasite cytochrome *bc*_*1*_ complex.

## Materials and methods

### 1. Parasite maintenance and transfection

*P*. *falciparum* D10 is the wildtype (WT) parasite line used in this study. D10attB-yDHOD was generated previously [27], which expresses the yeast DHOD gene of *Saccharomyces cerevisiae*. Parasites were cultured with RPMI 1640 medium (Invitrogen by Thermo Fisher Scientific) supplemented with 5 g/L Albumax I (Invitrogen), 10 mg/L hypoxanthine, 2.1 g/L sodium bicarbonate, HEPES (15 mM), and gentamycin (50 μg/mL). Cultures were maintained in human red blood cells (Type O, Interstate Blood Bank, Tennessee) and kept in a CO_2_/O_2_ incubator filled with a low oxygen mixture (5% O_2_, 5% CO_2_, and 90% N_2_). For transfections, ring stage parasites with 5% parasitemia (~ 300 μL) were mixed with plasmid DNA (50 μg in 50 μL) in cytomix buffer and electroporated using a Bio-Rad gene pulser. Drug medium (5 nM WR99210) was added 48 h post electroporation into the electroporated culture. The selectable marker on the knockout plasmid is hdhfr (human dihydrofolate dehydrogenase) [30].

### 2. Plasmid construction

1. Removal of yDHOD from the pAIO pre-gRNA construct. The pre-gRNA construct pAIO was generously provided by Dr. Josh Beck [31]; the plasmid contains yDHOD and *Streptococcus pyogenes* Cas9 coding sequences (CDS) connected by a 2A “self-cleaving” peptide. To remove yDHOD, pAIO was digested with BamHI and BglII to release the entire sequence of yDHOD and the first 250 bp of Cas9, since there is no unique restriction site between the two genes that could be used to release the yDHOD CDS alone. The first 250 bp of Cas9 were amplified from the original pAIO vector with primers P1 and P2, which include short homologous sequences that match the ends of the pAIO vector after its digestion with BamHI and BglII. The PCR product and the digested vector were then joined together using NEBuilder^®^ HiFi DNA Assembly (New England Biolabs^®^, Inc). A colony PCR was performed to screen colonies using primers P1 and P2. Positive clones were grown up, and their plasmid DNAs were digested with BamHI and BglII to confirm the loss of yDHOD. The positive plasmids were then sequenced using a primer upstream of Cas9 (P3) to confirm the intactness of Cas9. These procedures yielded the pre_gRNA construct without yDHOD, namely pAIO-Cas9-yDHOD(-).
2. PfNDH2 knockout construct. PfNDH2 (PF3D7_0915000) is 1602 bp long with no introns. We cloned the 5’ and 3’ homologous regions of PfNDH2 into a pCC1 vector bearing the hdhfr selectable marker [30]. The 5’HR (934 bp) was amplified with primers P4 and P5 and cloned into pCC1 digested by NcoI and EcoRI. Subsequently, the 3’HR (936 bp) was amplified with primers P6 and P7 and cloned into the vector digested by SpeI and SacII. After cloning, both 5’HR and 3’HR were sequenced (Genewiz LLC). The knockout construct was named 5'3'PfNDH2_pCC1. Maxi prep DNA of 5'3'PfNDH2_pCC1 (Qiagen) was digested with HincII overnight to linearize the vector before transfections.
3. Guide RNA constructs. The sequence between the 5’HR and 3’HR of PfNDH2 (490 bp) was submitted to the gRNA design tool (http://grna.ctegd.uga.edu/) to seek potential gRNAs. From the list of candidates, three sequences were chosen based on their high scores and zero off-target predictions. For each of these sequences, a pair of complementary oligonucleotides (60 or 61 bp) was synthesized and annealed in a mixture of NEB Buffers 2 and 4 by heating to 95°C for 5 minutes, then slowly cooling to room temperature. The vector, pAIO-Cas9-yDHOD(-), was digested with BtgZI and joined with the annealed oligonucleotide pair by gene assembly (New England Biolabs^®^, Inc), yielding a pAIO-Cas9-yDHOD(-)-gRNA construct. Other gRNA cloning procedures followed our published protocol [32].

Primers used for cloning procedures are listed below.

P1 (Remove yDHOD-F), 5’- ATACCTAATAGAAATATATCAGGATCCAAAAATGGACAAGAAGTACAGCATCG;
P2 (Remove yDHOD-R), 5’- CCATCTCGTTGCTGAAGATC;
P3 (Remove yDHOD-chk), 5’- GTATATTTTAAACTAGAAAAGGAATAAC;
P4 (knockout-5fF), 5’- gaccatggatatcaaaaaataatgcagtaaaatgc;
P5 (knockout-5fR), 5’- ccgaattCTGAACCTAGGATTATAATCTTTTCTTTTC;
P6 (knockout-3fF), 5’- ctactaGTGTCGAAGTTACCGCAGAATTTG;
P7 (knockout-3fR), 5’- aaccgcgGTCTTAATAAAATCGATGAAAAAATGGAACC;
P8 (gRNA1-F), 5’- CATATTAAGTATATAATATTgAATGTACCACTACATAAACAGTTTTAGAGCTAGAAATAGC;
P9 (gRNA1-R), 5’- GCTATTTCTAGCTCTAAAACTGTTTATGTAGTGGTACATTcAATATTATATACTTAATATG;
P10 (gRNA2-F), 5’- CATATTAAGTATATAATATTgCATGTAGCTGTTGTAGGAGGGTTTTAGAGCTAGAAATAGC;
P11 (gRNA2-R), 5’- GCTATTTCTAGCTCTAAAACCCTCCTACAACAGCTACATGcAATATTATATACTTAATATG;
P12 (gRNA3-F), 5’- CATATTAAGTATATAATATTgTTATTTAATTATAGCTGTAGGTTTTAGAGCTAGAAATAGC;
P13 (gRNA3-R), 5’- GCTATTTCTAGCTCTAAAACCTACAGCTATAATTAAATAAcAATATTATATACTTAATATG;
P14 (gRNA1-N20), 5’- AATGTACCACTACATAAACA;
P15 (gRNA2-N20), 5’- CATGTAGCTGTTGTAGGAGG;
P16 (gRNA3-N20), 5’- TTATTTAATTATAGCTGTAG;
P17 (N20CheckR), 5’- ATATGAATTACAAATATTGCATAAAGA;
P18 (5fchk), 5’- GAACTATACATCTATAAAGCATTAC;
P19 (3fchk), 5’- GAAAAAAGAAGCACATATATATATAT;
P20 (hDHFR-F), 5’- ATGCATGGTTCGCTAAACTGCATC;
P21 (hDHFR-R), 5’-ATCATTCTTCTCATATACTTCAAATTTGTAC.

### 3. Assessing parasite growth

PfNDH2 knockout and D10 WT lines were synchronized several times by alanine (0.5 M with 10 mM HEPES, pH 7.6) treatment. On day 0, parasites were inoculated into a 24 well plate with each well containing 2.5 mL of culture at 1% parasitemia and 3% hematocrit. Cultures were fed daily and split every two days. At each split (1:5), a sample of the parasitized RBCs was pelleted and fixed with 4% paraformaldehyde at 4°C overnight. After all samples were collected and fixed, they were washed with 1x PBS and stained with SYBR green I at 1:1000 (Catalog S7567, Life technologies by ThermoFisher Scientific). The samples were washed with PBS three times and analyzed on a C6 Flow Cytometer (BD). A total of 250,000 events were collected for each sample. Unstained infected RBCs and stained uninfected RBCs served as negative controls for gating. Growth curves were drawn using Graphpad Prism 6.

### 4. Growth inhibition assays using ^3^H-hypoxanthine incorporation

Inhibitor compounds were diluted by a series of three-fold dilutions in 96 well plates in low hypoxanthine medium (2.5 mg/L). Parasites were washed three times with low hypoxanthine medium, supplemented with fresh blood sufficient to make 1% parasitemia and re-suspended in the proper volume of low hypoxanthine medium to make a suspension with 3% hematocrit. Aliquots of the diluted culture were added to the 96-well plates containing the inhibitor dilution series in triplicates. After 24 h incubation, 10 μL of 0.5 μCi ^3^H-hypoxanthine was added to each well and the plates were incubated for another 24 h. After a total of 48 h incubation, the parasites were lysed by freezing-and-thawing, and nucleic acids were collected onto a filter using a cell harvester (Perkin Elmer). Radioactivity was counted using a Topcount scintillation counter (Perkin Elmer). In each condition, the percentage of growth (% growth) was determined by dividing radioactivity counts of drug treated wells by control wells with no drug treatment. Data were analyzed and graphed using Graphpad Prism 6.

### 5. Ubiquinol-cytochrome *c* reduction assay

Mitochondria of ΔPfNDH2 and D10 WT were individually isolated using a method published previously [32, 33]. Briefly, a large volume of parasite culture of each line (~2 liters) was lysed with saponin (0.05%) and disrupted in a N_2_ cavitation chamber (4639 Cell Disruption Vessel, Parr Instrument Company) in an isotonic mitochondrial buffer. The total parasite lysate was spun down at 900 *g* for 6 min to remove large debris, and the cloudy supernatant was passed through a MACS CS column (Miltenyi Biotec) in a Vario MACS magnetic separation apparatus to remove most of the hemozoin, according to our published protocol [33]. The eluted light yellow material was pelleted at 23,000 × *g* for 20 min at 4 °C, and the pellet was re-suspended in buffer and stored at -80 °C. The cytochrome *c* reductase activity of the *bc*_1_ complex was measured with a modification of previous methods [32–34]. The assay volume was 300 μL, containing mitochondrial proteins (25 μg), 100 μM decylubiquinol (reduced), 75 μM horse heart cytochrome *c* (Sigma-Aldrich), 0.1 mg/mL n-docecyl-β-D-maltoside, 60 mM HEPES (pH 7.4), 10 mM sodium malonate, 1 mM EDTA, and 2 mM KCN, and was incubated at 35°C in a stirred cuvette. Reduction of oxidized horse heart cytochrome *c* was recorded at 550 nm with a CLARITY VF integrating spectrophotometer (OLIS, Bogart, GA). A Bio-Rad colorimetric assay was used to measure protein concentrations of all mitochondrial samples. For each mitochondrial sample, the maximal activity of ubiquinone-cytochrome *c* reduction (100%) was averaged from five measurements as described above with no addition of any inhibitors. Compounds were dissolved in DMSO and tested in a series of concentrations with each concentration in two or three replicates. The concentration varied from 0.003 nM to 2.5 μM for HDQ and from 0.001 nM to 15.6 μM for CK-2-68. Note, n-docecyl-β-D-maltoside was used as a detergent in the assay.

### 6. NADH-cytochrome *c* reductase assay

Assay conditions were similar to those described above for the ubiquinol-cytochrome *c* reduction assay. The 300 μL assay mix contained 25 μg of mitochondrial proteins, 50 μM oxidized horse heart cytochrome *c*, 60 mM HEPES (pH 7.4), 10 mM sodium malonate, 1 mM EDTA, 2 mM KCN and 300 μM NADH. In this assay, the reduced decylubiquinol was replaced with NADH to provide electrons for reducing horse heart cytochrome *c*. As described above, reduction of horse heart cytochrome *c* was recorded at 550 nm with a CLARITY VF integrating spectrophotometer (OLIS, Bogart, GA). The assay buffer contained no detergent, since it was reported that detergents heavily interfere with assays of NADH oxidation [35]. For each condition, data was averaged from three replicates (n = 3).

## Results

### PfNDH2 is not essential in asexual blood stages of *Plasmodium falciparum*

**Fig 1:**
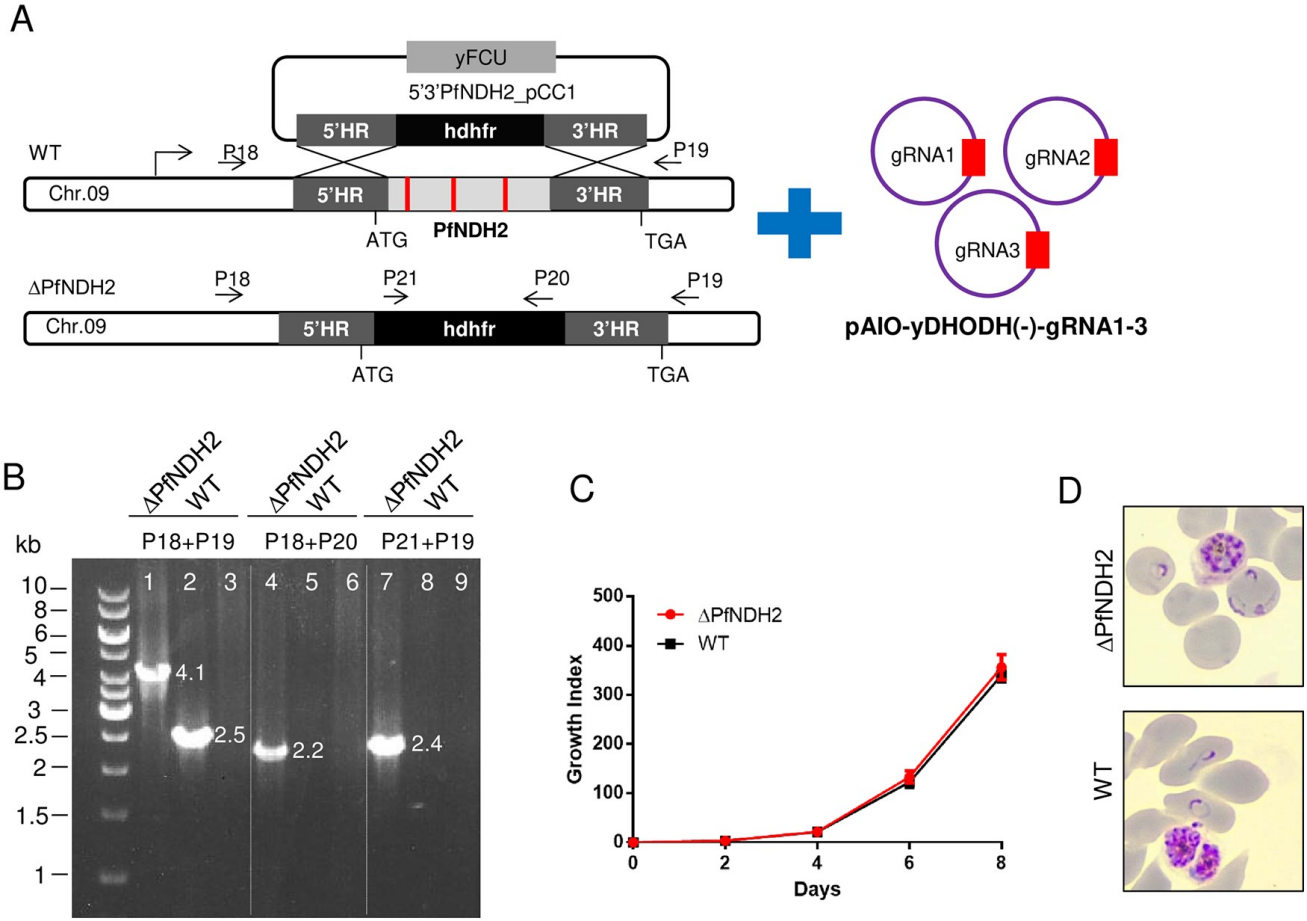
Disruption of the type II NADH dehydrogenase of *P*. *falciparum* does not affect growth in asexual blood stages. (A) A schematic diagram depicts the genetic deletion of a large segment of PfNDH2 via CRISPR/Cas9-assisted homologous recombination. The knockout plasmid (5’3’PfNDH2_pCC1) was co-transfected with three gRNA constructs made in pAIO-Cas9-yDHOD(-) (see Materials and methods). 5’HR, 5’ homologous region; 3’HR, 3’ homologous region. yFCU, yeast fusion gene of cytosine deaminase and uracil phosphoribosyl transferase. hdhfr, human dihydrofolate dehydrogenase. ATG is the start codon of PfNDH2 gene and TGA is the stop codon. (B) A diagnostic PCR confirming the genotype of the ΔPfNDH2 parasite. Primer positions are shown in (A). In ΔPfNDH2, a 4.1 kb knockout band (Lane 1), a 2.2 kb 5’ integration band (Lane 4) and a 2.4 kb 3’ integration band (Lane 7) were detected. In WT, only a 2.5 kb band was detected (Lane 2) whereas no 5’ integration (Lane 5) or 3’ integration (Lane 8) was observed. Lanes 3, 6, and 9 were negative controls with no DNA in PCR reactions. (C) A growth curve of the ΔPfNDH2 parasite determined by SYBR green staining and flow cytometry analysis (Materials and methods). (D) The ΔPfNDH2 parasite is morphologically healthy. Representative Giemsa stained thin smears of WT and ΔPfNDH2 cultures are shown displaying schizont and ring forms.

Transcriptomics data indicate that the type II NADH dehydrogenase in *P*. *falciparum* (PF3D7_0915000) is expressed in the asexual blood stages (PlasmoDB.org). It has been shown that the leader sequence of PfNDH2 was able to target GFP into the mitochondrion [36], suggesting that PfNDH2 is a mitochondrial enzyme. To further confirm that, we genetically tagged PfNDH2 with 3x HA (hemagglutinin) and the tagged PfNDH2 was localized to the parasite mitochondrion by immunofluorescence assays [37]. To assess the essentiality of PfNDH2, we employed the CRISPR/Cas9 DNA repair technique. A knockout plasmid vector was constructed, containing a 5’HR (homologous region) mostly upstream of the gene’s coding sequence (CDS) (outside) and a 3’HR near the end of the CDS (inside) (Fig 1A, Materials and methods). The 3’HR was chosen from the coding region to circumvent inclusion of overly high AT content in the knockout vector. Three gRNA sequences targeting the PfNDH2 gene were individually cloned into a modified Cas9 plasmid, pAIO-Cas9-yDHOD(-), from which yDHOD had been removed (Materials and methods). Previous studies have shown that expression of yDHOD in malaria parasites renders the entire mtETC nonessential by providing a metabolic bypass for pyrimidine biosynthesis [1]. Therefore, to assess the essentiality of PfNDH2 in the context of a normal mtETC, we removed yDHOD from the gRNA vectors. The knockout plasmid was linearized by restriction digestion and transfected into D10 WT parasites together with all three circular gRNA vectors (Materials and methods). Viable transgenic parasites were observed under WR99210 selection three weeks post transfection. A diagnostic PCR revealed that the genomic locus of PfNDH2 was disrupted (Fig 1B). We then tightly synchronized both ΔPfNDH2 and WT lines and examined the growth rates over 4 intraerythrocytic developmental cycles (IDCs) via flow cytometry. ΔPfNDH2 parasites did not show any noticeable grow defects in comparison to WT parasites (Fig 1C). Representative Giemsa-stained images of ΔPfNDH2 and WT parasites were shown, suggesting that deletion of PfNDH2 also did not appear to affect parasite health and morphology (Fig 1D). Collectively, our data show that PfNDH2 is not essential in asexual blood stages of *P*. *falciparum*, consistent with the knockout study carried out previously in the rodent malaria parasite, *P*. *berghei* [28]. These results argue against the long-held assumption that PfNDH2 is an attractive drug target [9–11, 18].

### The ΔPfNDH2 parasite is equally susceptible to mtETC inhibitors

**Fig 2:**
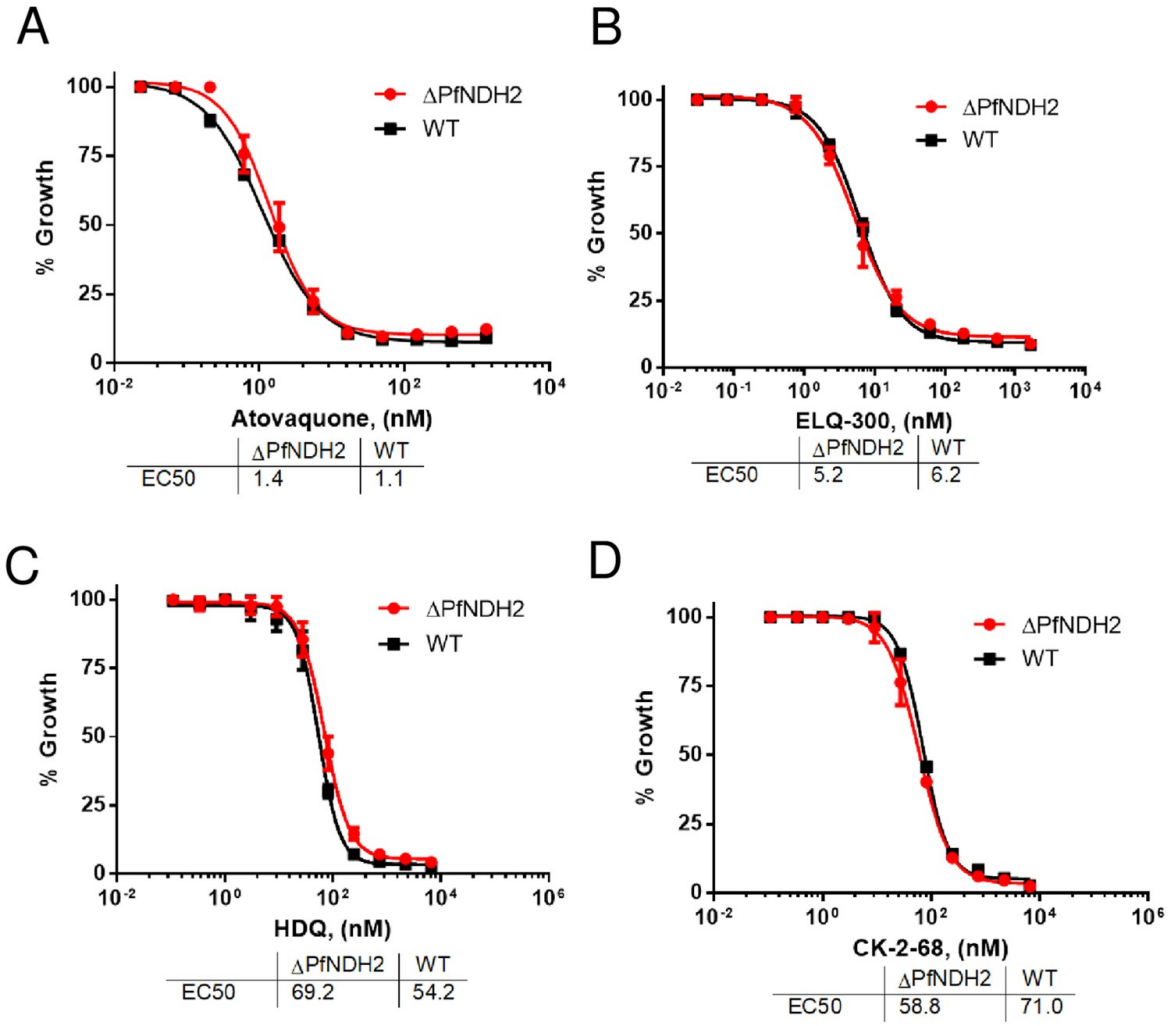
Deletion of PfNDH2 does not affect sensitivity to mitochondrial electron transport chain inhibitors. ^3^H-hypoxanthine incorporation assays were performed in the ΔPfNDH2 and WT parasites challenged with atovaquone (A), ELQ-300 (B), HDQ (C) and CK-2-68 (D). For each compound in each parasite line, a mean EC_50_ value of replicated experiments is shown at the bottom of each panel. Data shown is a representative of n≥3 replicates. Error bars indicate standard deviations of three technical replicates in the representative assay.

The healthy growth of the ΔPfNDH2 parasites *in vitro* suggests that the parasite mtETC remains functionally competent in the absence of PfNDH2 (Fig 1). To challenge the knockout parasites, we exposed them to mtETC inhibitors in growth inhibition assays, measured as ^3^H-hypoxanthine incorporation. In comparison to the WT, the ΔPfNDH2 parasites were equally sensitive to atovaquone (a Q_o_ site inhibitor of the *bc*_*1*_ complex) and ELQ-300 (a Q_i_ site inhibitor) [8] (Fig 2a+2b). Thus, these data suggest that deletion of PfNDH2 has little effect on the sensitivity of asexual parasites to downstream inhibitors of the mtETC. The loss of PfNDH2, thus, does not appear to affect the function of the remainder of the mtETC. As noted previously, HDQ and its newer derivative CK-2-68 were considered to be PfNDH2 specific inhibitors [21–23] or, more recently, dual-targeting inhibitors of cytochrome *bc*_*1*_ as well as PfNDH2 [38]. In that case, HDQ and CK-2-68 would be expected to lose potency, at least partially, in the ΔPfNDH2 parasite, since the putative primary target is absent. However, HDQ and CK-2-68 were still highly potent in the knockout parasite as shown above, suggesting that HDQ and CK-2-68 primarily target other sites than PfNDH2 (Fig 2c+2d).

### The *bc*_*1*_ complex of the mtETC is the target of HDQ and CK-2-68

**Fig 3:**
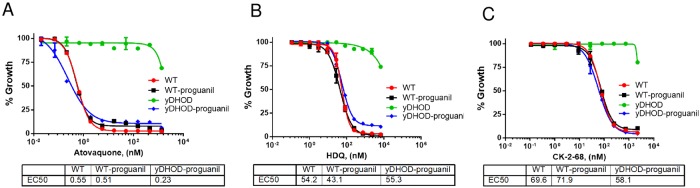
HDQ and CK-2-68 target the *bc*_*1*_ complex. D10attB-yDHOD transgenic (yDHOD) or wildtype (WT) parasites were challenged with atovaquone (A), HDQ (B) and CK-2-68 (C) with and without proguanil (1 μM). Growth was measured using ^3^H-hypoxanthine incorporation assays. For each compound, a mean EC_50_ value is shown at the bottom of each panel for WT parasites (with or without proguanil) and yDHOD parasites treated with proguanil. (Note, EC_50_ values are not valid in yDHOD parasites when the parasites are fully resistant to compounds in test.) Data shown is a representative of n = 3 replicates. Error bars show standard deviations of three technical replicates in the representative assay.

Our data above suggests that HDQ and CK-2-68 target an activity other than that of PfNDH2 (Fig 2). Since HDQ and CK-2-68 are ubiquinone analogs, we suggest that they kill malaria parasites by targeting the *bc*_*1*_ complex, although Vallieres *et al*. and Biagini *et al*. previously suggested that HDQ and CK-2-68 had a dual effect on both PfNDH2 and the *bc*_*1*_ complex [38, 39]. To distinguish between these alternatives, we performed growth inhibition assays in the yDHOD transgenic parasite line (in D10attB) using HDQ and CK-2-68 in combination with proguanil. As shown previously, expression of the yDHOD gene bypasses the need for mtETC function in asexual parasites [1]. The yDHOD transgenic parasites have become a handy tool to examine whether a compound targets the mtETC, as all mtETC inhibitors suffer a large loss of potency in the yDHOD background, which applies to both *bc*_*1*_ inhibitors and PfDHOD inhibitors [40]. Further, a low concentration of proguanil can restore sensitivity to *bc*_*1*_ inhibitors in yDHOD transgenic parasites [1], but not for PfDHOD inhibitors. Proguanil has an EC_50_ of ~60 μM in WT parasites [1]. One μM proguanil was used in the assay to verify if parasites became hypersensitive to proguanil. As a control, we showed that yDHOD parasites were fully resistant to atovaquone but became fully sensitive in the presence of 1 μM proguanil (Fig 3). Upon inhibition by atovaquone, the yDHOD parasites lose their primary source of generating mitochondrial membrane potential (ΔΨ_m_), conveyed by the *bc*_*1*_ complex and cytochrome *c* oxidase of the mtETC, and become hypersensitive to proguanil, which likely targets a secondary generator of ΔΨ_m_ (1). Using this system, we tested the HDQ and CK-2-68 sensitivity of the yDHOD parasites with and without 1 μM proguanil. As expected, yDHOD parasites were highly resistant to HDQ and CK-2-68; upon proguanil treatment, however, the yDHOD parasites regained sensitivity to these compounds (Fig 3). HDQ and CK-2-68, thus, behaved in a very similar manner to atovaquone against the yDHOD transgenic parasites, indicating that HDQ and CK-2-68 target the *bc*_*1*_ complex. These results are consistent with a previous report that found parasites grow normally in the presence of 10 μM HDQ when expressing the yDHOD gene [41]. Furthermore, recent chemical mutagenesis experiments using CK-2-68 generated mutations that were all in the *cyt b* locus, rather than in PfNDH2 [29]. Collectively, these results indicate that HDQ and CK-2-68 are potent cytochrome *bc*_*1*_ inhibitors. Thus, our data here do not support the dual targeting effect of HDQ and CK-2-68 against both PfNDH2 and the *bc*_*1*_ complex, as previously suggested [38, 39].

### HDQ and CK-2-68 directly inhibit the enzymatic activity of the *bc*_*1*_ complex

**Fig 4:**
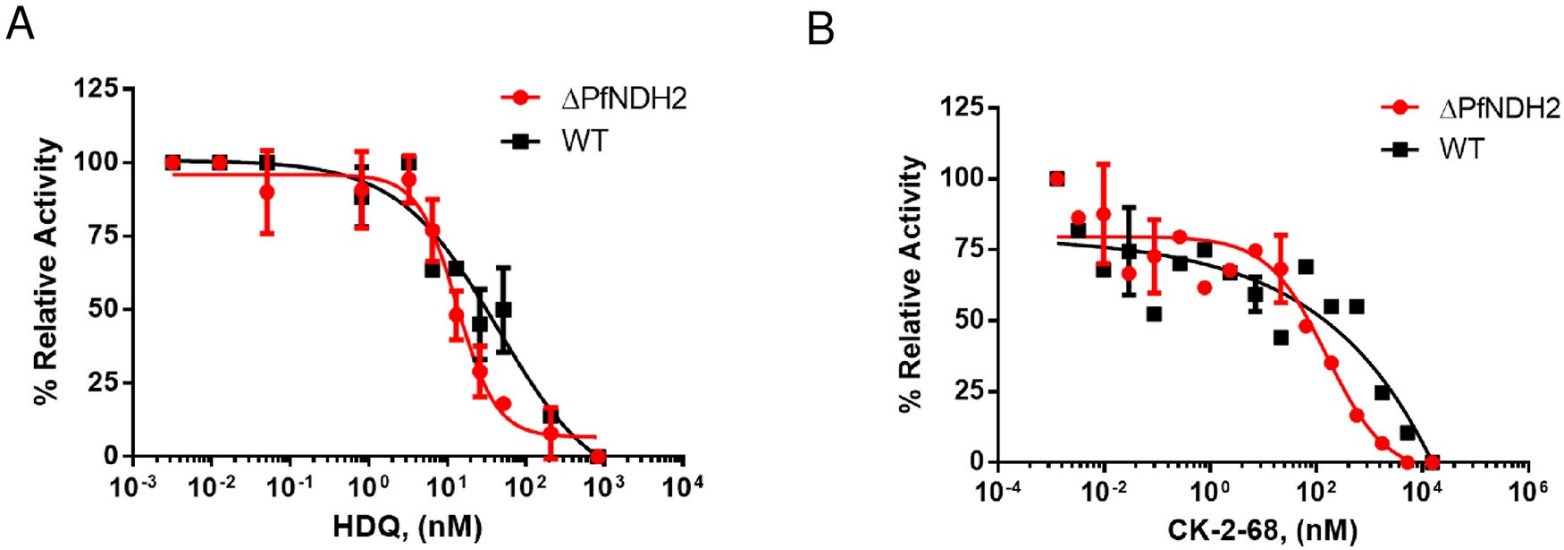
HDQ and CK-2-68 directly inhibit mitochondrial ubiquinol-cytochrome *c* reductase activity *in vitro*. In each measurement, the same amount of mitochondria of knockout or WT (25 μg of sample) was used. Reduction of cytochrome *c* was followed spectrophotometrically at 550 nm (Materials and methods). The rates of reduction in the presence of various concentrations of inhibitors were normalized to that of no drug controls (average of 5 replicates), resulting in relative activity (%). (A) Inhibition by HDQ. Data shown is plotted from n = 3 biological replicates. (B) Inhibition by CK-2-8. Data shown is a representative of n = 2 biological replicates. Error bars indicate standard deviations of technical replicates (two or three) in the representative assay.

In addition to growth inhibition assays as described above, we also directly investigated the effect of HDQ and CK-2-68 on the enzymatic activity of the *bc*_*1*_ complex in a preparation enriched in parasite mitochondria using a spectrophotometric assay (Materials and methods) [33]. HDQ and CK-2-68 inhibited the ubiquinol-cytochrome *c* reductase activity in the mitochondria of ΔPfNDH2 and WT in a dose dependent manner (Fig 4). Importantly, the inhibitory potency of HDQ and CK-2-68 were equally robust in two types of mitochondria from WT and ΔPfNDH2, respectively. This provides further evidence that the antimalarial mode of action of HDQ and CK-2-68 arises from inhibition of the *bc*_*1*_ complex, rather than PfNDH2.

### *In vitro* measurement of NADH linked cytochrome *c* reductase activity

**Fig 5:**
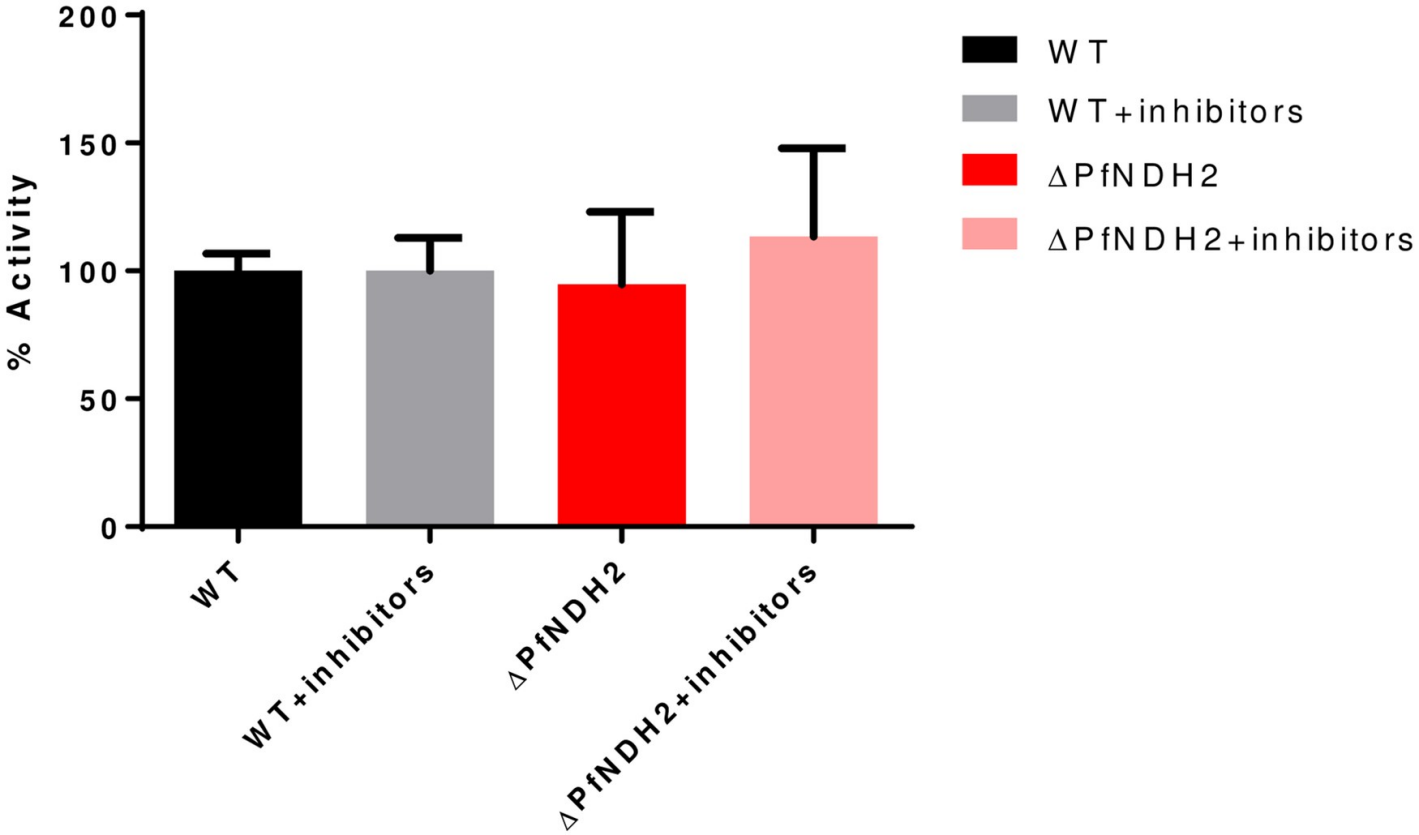
NADH-linked cytochrome *c* reductase activity in the *in vitro* assay is not dependent on PfNDH2 or mtETC. In each measurement, 25 μg of mitochondrial sample was used. Rates of cytochrome reduction were measured without and with addition of a mix of *bc*_*1*_ inhibitors, including atovaquone (62 nM), ELQ-300 (62 nM) and HDQ (3,100 nM). The concentrations of these inhibitors were equal or greater than 100 times of their individual EC_50_ values. Data shown is mean ± s.d. of n = 3 replicates.

Previously data from Fry and Beesley revealed a relatively strong NADH-cytochrome *c* reductase activity using NADH as a reductant in parasite mitochondrial preparations *in vitro* [19]. Interestingly, the activity was not inhibited by rotenone at 80 μM or antimycin A at 20 μM (a Qi inhibitor) [19]. Rotenone insensitivity suggested that malaria parasites lack a conventional multi-subunit Complex I, which was later confirmed by genome sequencing project [42]. The reason why antimycin A did not inhibit the NADH-cytochrome *c* reductase activity in Fry and Beesley’s assays has been elusive. Antimycin A did effectively inhibit cytochrome *c* reductase activity when other mitochondrial reductants were used, such as α-glycerophosphate and succinate (19). Later on, antimycin A was found to kill malaria parasites in whole cell assays with an EC_50_ of just 13 nM [43]. Clearly, antimycin A was a robust inhibitor of the parasite mtETC. Hence, the provenance of a strong NADH-cytochrome *c* reductase activity independent of an mtETC inhibitor observed in mitochondrial preparations has been an unsettled issue [19]. In intact parasites, NADH oxidized by NDH2 is presumed to pass electrons to ubiquinone, which are then transferred on to the *bc*_*1*_ complex, cytochrome *c*, cytochrome *c* oxidase and, finally, to O_2_. If the *in vitro* assay were replicating the initial steps of the *in vivo* pathway, we should observe a much diminished NADH-cytochrome *c* reductase activity using NADH as a reductant in the ΔPfNDH2 parasites since PfNDH2, missing in the knockout parasite, is the only known enzyme donating electrons from NADH to the mtETC in the parasites [14]. However, deletion of PfNDH2 had no effect on NADH-cytochrome *c* reductase activity measured at 550 nm when NADH was used as a reductant, suggesting that this *in vitro* assay is not linked to PfNDH2 (Fig 5). Further, in both ΔPfNDH2 and WT mitochondrial samples, NADH-cytochrome *c* reductase activity was not inhibited by a mix of malaria parasite specific *bc*_*1*_ inhibitors, including atovaquone (62 nM), ELQ-300 (62 nM), and HDQ (3,100 nM), each at equal or greater than 100x EC_50_ (Fig 5). Thus, our data are consistent with the earlier observation that antimycin A failed to inhibit the NADH-cytochrome *c* reductase assay [19] and suggest that the *in vitro* NADH-cytochrome *c* reductase activity is likely non-enzymatic (see Discussion).

## Discussion

A common strategy for developing antimicrobial drugs is to target divergent proteins of the microbe to circumvent potential toxicity against the host. Proteins unique to microbes are even more interesting as their inhibitors would potentially have little to no side effects in the host. The type II NADH dehydrogenase is present in malaria parasites but not in humans; thus, it has been considered an attractive prospective drug target for a long time [9, 11, 44]. However, a unique protein may not necessarily be an essential one. A valid drug target should normally be essential to the pathogen in order that its inhibition will arrest growth and/or kill the pathogen. Initial failures to disrupt the NDH2 gene in *P*. *falciparum* parasites suggested that the gene might be essential [35, 37]. On the other hand, data on the effect of mtETC inhibitors in yDHOD transgenic parasites [1] and the reported knockout of NDH2 in *P*. *berghei* [28] suggested that PfNDH2 should be dispensable in asexual parasites (as described above). Without conclusive data, however, it remains an unsettled issue in the field whether PfNDH2 is a good antimalarial drug target. In this report, we have provided evidence that PfNDH2 is dispensable in asexual blood stages and, therefore, is unlikely to be an effective antimalarial drug target. We note that our knockout result is consistent with the recent genetic screen of *P*. *falciparum* growth phenotypes, in which a PiggyBac transposon insertion was recovered in the gene coding region of PfNDH2, suggesting non-essentiality of this gene [45].

Our results suggest that the parasite mtETC is functionally intact in the absence of PfNDH2. Not only is the growth of the knockout line closely similar to that of the WT parental line, but the response to cytochrome *bc*_*1*_ inhibitors is virtually identical (Figs 1 and 2). Evidently, in the ΔPfNDH2 parasites, the other ubiquinone-dependent dehydrogenases—MQO, SDH, G3PDH, and DHOD—supply sufficient amount of ubiquinol to maintain adequate function of the mtETC during asexual development. DHOD is essential for the parasite’s pyrimidine *de novo* synthesis pathway, since malaria parasites cannot salvage pyrimidine precursors [46]. Other dehydrogenases of the mtETC, however, may be functionally redundant as electron donors. In support of this, the flavoprotein (Fp) of SDH in *P*. *berghei* was deleted without an effect on parasite growth in asexual blood stages [47]. Likewise, a recent study of *P*. *berghei* MQO suggested that PbMQO was not essential for asexual growth although a delayed growth rate was observed in the knockout parasite [48]. Furthermore, the recent genome-wide knockout study in *P*. *berghei* also suggested that G3PDH was dispensable in asexual blood stages (plasmogem.sanger.ac.uk). Genetic data is needed to individually verify the essentiality of MQO, SDH and G3PDH in *P*. *falciparum*. The recent large-scale genetic mutagenesis screening performed in *P*. *falciparum* with PiggyBac has suggested that PfMQO was dispensable whereas PfSDH (flavoprotein) and PfG3PDH had a low mutagenesis score (with essentiality uncertain) [45].

Although HDQ and CK-2-68, and probably other related derivatives (29) do not primarily target PfNDH2 in parasites, as shown by our results, they are potent antimalarial compounds via inhibition of the parasite *bc*_*1*_ complex. Importantly, HDQ and CK-2-68 retained their potency in atovaquone resistant parasites [38, 39]. Experiments with yeast *cyt b* mutants suggested that HDQ likely bound to the Qi site of *bc*_*1*_ complex, whereas atovaquone is a Qo site inhibitor [38]. CK-2-68, on the other hand, is likely to be a Qo site inhibitor, but, nevertheless, exhibited no cross resistance with atovaquone [29]. Biagini *et al*. have developed additional quinolone derivatives with more favorable pharmacological properties that were predicted to bind at the Qo site (39). Combinations of non-cross resistant *bc*_*1*_ inhibitors may be effective at slowing the development and spread of resistance, since strong resistance mutations in *cyt b* may exert a significant survival fitness cost [49], including blocking transmission [50]. Thus, the development of additional antimalarial candidates targeting the *bc*_*1*_ complex may facilitate the future development of effective combination therapies. Indeed, atovaquone and ELQ-300, Q_o_ and Q_i_ inhibitors respectively, were recently shown to be a highly effective and synergistic antimalarial combination [51].

The results of our attempts to measure *in vitro* NADH-cytochrome *c* reductase activity spectrophotometrically provide a cautionary tale for the design and interpretation of assays involving the oxidation of NADH *in vitro*, a reactive reductant. Neither elimination of PfNDH2 nor strong inhibition of the cytochrome *c* reductase activity of *bc*_*1*_ affected the observed reaction, implying that the reaction does not proceed through the pathway of mtETC (Fig 5). Fry and Beasley apparently observed the same phenomenon when they measured apparent NADH-cytochrome *c* reductase activity in *Plasmodium* mitochondria with and without antimycin A, a general Qi site inhibitor of the *bc*_*1*_ complex [19]. Given the report that detergents (which form micelles) accelerate NADH oxidation [35], we speculate that it may be the presence of mitochondrial phospholipid membranes in the mitochondrial samples that produce this effect. Cytochrome *c* is known to bind to phospholipids head groups [52], so mitochondrial particles could provide a surface that concentrates cytochrome *c* for reaction with NADH for a direct reaction without any aid from an enzyme. The non-enzymatic reaction may also be facilitated by the relatively high concentration of cytochrome *c* used in spectrophotometric assays (50–100 μM). At any rate, our results demonstrate that the apparent robust NADH-cytochrome *c* activity that has been reported in *Plasmodium* mitochondrial preparations *in vitro* [19] is not an indication of high NADH dehydrogenase activity in intact parasites *in vivo*.

In summary, our study implies that PfNDH2 is not essential in asexual blood stages therefore it is not likely a good antimalarial drug target. Although PfNDH2 is still likely required for malaria transmission [28], inhibition of PfNDH2 is expected to have no effect on parasite growth in asexual blood stages.
